# Success of ^177^Lu-DOTATATE therapy in a metastatic pituitary neuroendocrine tumor

**DOI:** 10.1530/EO-25-0073

**Published:** 2025-10-18

**Authors:** Katherine I Wolf, Zhonglin Lu, Elizabeth A Hesseltine, Ka-Kit Wong, Francis P Worden, Tobias Else

**Affiliations:** ^1^ Department of Internal Medicine, Division of Metabolism, Endocrinology & Diabetes, Michigan Medicine, Ann Arbor, Michigan, USA; ^2^ Department of Radiology, Division of Nuclear Medicine, Michigan Medicine, Ann Arbor, Michigan, USA; ^3^ Department of Internal Medicine, Division of Hematology and Oncology, Michigan Medicine, Ann Arbor, Michigan, USA

**Keywords:** pituitary neuroendocrine tumor, ^177^Lu-DOTATATE, PRRT, Cushing syndrome, dosimetry

## Abstract

**Abstract:**

Metastatic pituitary neuroendocrine tumors (PitNETs) are rare and often aggressive. Patients should be evaluated for metastatic disease when their biochemical presentation is discordant with pituitary tumor burden or when they present with non-pituitary, site-specific symptoms. Here, we describe the case of a 55-year-old male with an ACTH-secreting PitNET and more than a decade of recurrent transsphenoidal resections, gamma knife irradiation, and therapy with somatostatin analogs and steroidogenesis inhibitors before uncovering metastatic osseous deposits. Only after initiation of peptide receptor radionuclide therapy (PRRT) with ^177^Lu DOTATATE has he had sustained clinical, biochemical, and structural improvement. There are a limited number of cases of patients with metastatic PitNET who underwent PRRT, but the majority have been favorable with limited adverse events, including hematologic cytopenias, and tumor stability and/or shrinkage. While there are several other off-label, novel therapies used in this population, including non-endocrine targeted therapies or immunotherapy, further studies should evaluate the earlier introduction of PRRT in metastatic PitNET given the good safety profile and overall clinical success.

**Learning points:**

- Patients with aggressive, recurrent PitNET and discordant biochemical and structural data or site-specific symptoms should be evaluated for evolution of disease, including metastases.
- There are multiple alternative agents beyond cytotoxic chemotherapy for progressive metastatic PitNETs, including non-endocrine targeted therapies, immunotherapy, and peptide receptor radionuclide therapy (PRRT).
- PRRT has successfully been used in patients with metastatic PitNET without significant adverse events while stabilizing tumor burden and clinical hormone excess.

## Background

In 2022, the World Health Organization reclassified pituitary ‘carcinoma’ as metastatic pituitary neuroendocrine tumor (PitNET). PitNETs are typically indolent, with aggressive lesions metastasizing in just 0.13–0.4% of cases (Villa *et al.* 2023). Patients with known aggressive PitNETs and non-pituitary, site-specific symptoms or discordant imaging and biochemistry (increase in hormone levels without correlative tumor growth) should be evaluated for metastases (Raverot *et al.* 2025). Histopathology has been studied as a predictor of an aggressive or metastatic course, but results vary; however, a Ki-67 proliferative index >3% is felt to indicate a high risk of recurrence (Raverot *et al.* 2025). While this remains controversial without prospective validation, it demonstrates the statistical power to predict PitNET regrowth (Petry *et al.* 2019).

Given the rarity of metastatic PitNET, a multidisciplinary treatment plan is necessary, with consideration for re-resection, re-irradiation, hormonal therapies, and cytotoxic chemotherapies (Robertson *et al.* 2023, Raverot *et al.* 2025). To date, targeted treatment for VEGF/VEGF receptor, immunotherapy, or peptide receptor radionuclide therapy (PRRT) has shown promise but is not standard of care (Heaney 2011, Burman *et al.* 2023, Robertson *et al.* 2023). Here, we report the case of a patient with osseous PitNET metastases more than a decade after his original presentation with biochemical and structural improvement following PRRT with ^177^Lu DOTATATE therapy.

## Case presentation

A 38-year-old male with a 5-year clinical history of classic Cushing’s disease underwent transsphenoidal resection of a 2.5 × 2.3 cm pituitary macroadenoma followed by gamma knife ablation complicated by secondary hypothyroidism and hypogonadism. Two years later, he developed recurrent hypercortisolism and was initiated on ketoconazole after a failed second course of gamma knife irradiation. He remained overtly cushingoid with poorly controlled multi-agent hypertension (HTN), prompting elective laparoscopic bilateral adrenalectomy. Shortly thereafter, repeat magnetic resonance (MR) imaging demonstrated significant growth of the residual tumor, prompting a third round of gamma knife irradiation. Several years later, following persistently elevated ACTH, continued tumor growth, and concern for Nelson syndrome, he was initiated on cabergoline without improvement and quickly transitioned to somatostatin analogs (SSAs). He then opted to pursue repeat transsphenoidal resection and a fourth round of gamma knife therapy. Post-operatively, he had persistent ACTH elevation but without correlate on anatomic imaging and was conservatively managed for several years.

Fifteen years after his initial diagnosis, he presented to a local emergency department for back pain and was incidentally found to have several 3-cm marrow-replacing intramedullary lesions of the right proximal femoral shaft and left acetabulum with perilesional bone marrow edema and periosteal reaction. Core needle biopsy showed fibrosis and reactive-appearing spindle cells with focal bone formation consistent with fracture callus, and he was discharged with instructions to follow up with an orthopedist and endocrinologist.

## Investigation

**Figure 1:**
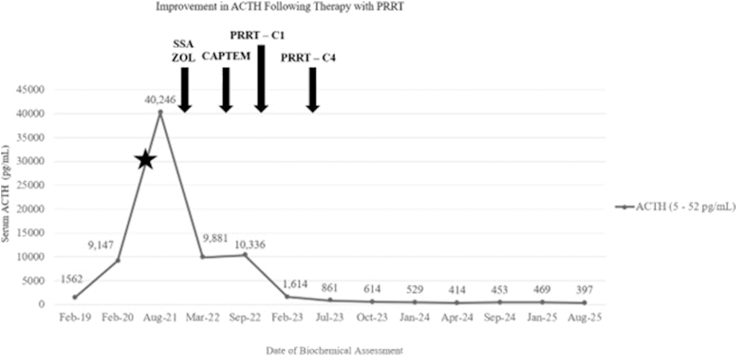
Serum ACTH levels as the patient progressed with therapy, including the SSA lanreotide (120 mg SQ every 4 weeks) and zoledronic acid (4 mg/100 mL IV every 3 months), cytotoxic chemotherapy with CAPTEM, and PRRT (200 mCi ^177^Lu DOTATATE every 8 weeks for four cycles). The star represents discovery of metastatic bone lesions. Abbreviations: C (cycle); CAPTEM (capecitabine/temozolomide); IV (intravenous); Lu (lutetium); mCi (millicurie); PRRT (peptide receptor radionuclide therapy); SSA (somatostatin analog); SQ (subcutaneous); ZOL (zoledronic acid).

Following identification of his bone lesions, laboratory testing demonstrated a significant increase in ACTH as high as 40,246 pg/mL (8,862 pmol/L) (reference range (RR) 5–52 pg/mL; 2–11 pmol/L) and a chromogranin A (CgA) peak of 138 ng/mL (310 pmol/L) (RR < 93 ng/mL; <60 pmol/L) from 9,147 pg/mL (2,012 pmol/L) and 91 ng/mL (204 pmol/L) 2 years prior, respectively (Fig. 1). Complete blood count, comprehensive metabolic panel, prostate-specific antigen, and sedimentation rate were unremarkable.

Functional imaging with ^68^Ga DOTATATE positron emission tomography (PET) showed avid uptake in the parasellar region extending along the skull base with osseous metastases involving the left parietal bone, left acetabulum, and right femoral shaft. Repeat computed tomography (CT)-guided core biopsy of the left acetabulum indicated metastatic ACTH-positive, well-differentiated neuroendocrine carcinoma consistent with metastases from a corticotroph pituitary primary.

## Treatment

He was initiated on subcutaneous, monthly lanreotide (60 mg) and intravenous zoledronic acid (4 mg) for bone pain related to known metastatic deposits. Following 6 months of therapy, ACTH and CgA decreased to 9,881 pg/mL (2,173 pmol/L) and 34 ng/mL (76 pmol/L), respectively. However, repeat MR brain showed an increase in the left parasellar mass with extension to the prepontine cistern, resulting in mass effect on the pons and cerebellar peduncle.

He was briefly started on capecitabine and temozolomide with continuation of the above regimen while he was evaluated for PRRT with ^177^Lu DOTATATE. Following two cycles of chemotherapy, he received 200 mCi ^177^Lu DOTATATE every 8 weeks for a total of four cycles.

## Outcome and follow-up

After ^177^Lu DOTATATE administration, post-therapy quantitative single-photon emission computed tomography (SPECT/CT) scans were obtained. For cycles 1–3, multi-timepoint SPECT/CT-based dosimetry was performed using MIM Software Inc. (Dewaraja *et al.* 2022). Sequential SPECT/CT images were co-registered, followed by Monte Carlo radiation transport to generate absorbed dose (AD) rate maps, which were fit with mono- or bi-exponential functions based on the number of timepoints and Akaike criteria and then integrated to yield an AD. For cycle 4, single-timepoint dosimetry was performed using prior information from cycle 3 (Del Prete *et al.* 2018).

**Figure 2:**
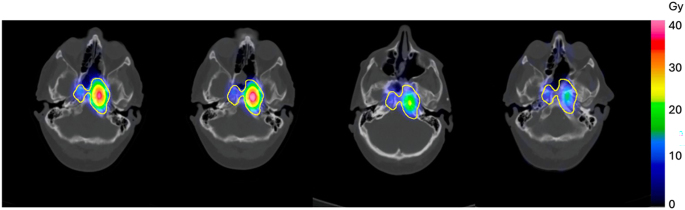
AD map of the index lesion from cycles 1 – 4 (left to right) during therapy with ^177^Lu DOTATATE PRRT every 8 weeks. A declining trend in mean lesion AD was observed with values of 2.14, 2.13, 1.41, and 1.05 Gy/GBq from cycle 1 to 4, respectively, especially from cycle 2 to 4, indicating likely treatment response. Abbreviations: GBq (gigabecquerel); Gy (gray); Lu (lutetium); PRRT (peptide receptor radionuclide therapy).

A declining trend in mean lesion AD was observed from cycle 1 to 4 (Fig. 2), with values of 2.14, 2.13, 1.41, and 1.05 Gy/GBq, respectively. Meanwhile, ADs to non-target organs remained relatively constant across cycles – left kidney (0.54, 0.59, 0.51, and 0.58 Gy/GBq), right kidney (0.56, 0.62, 0.52, and 0.61 Gy/GBq), spleen (0.61, 0.74, 0.60, and 0.65 Gy/GBq), and liver (0.09, 0.09, 0.10, and 0.09 Gy/GBq). The cumulative mean AD to the kidneys was 17.14 Gy (volume-weighted average of the left and right kidneys), which remained below the recommended dose limit of 23 Gy (Dewaraja *et al.* 2022).

**Figure 3:**
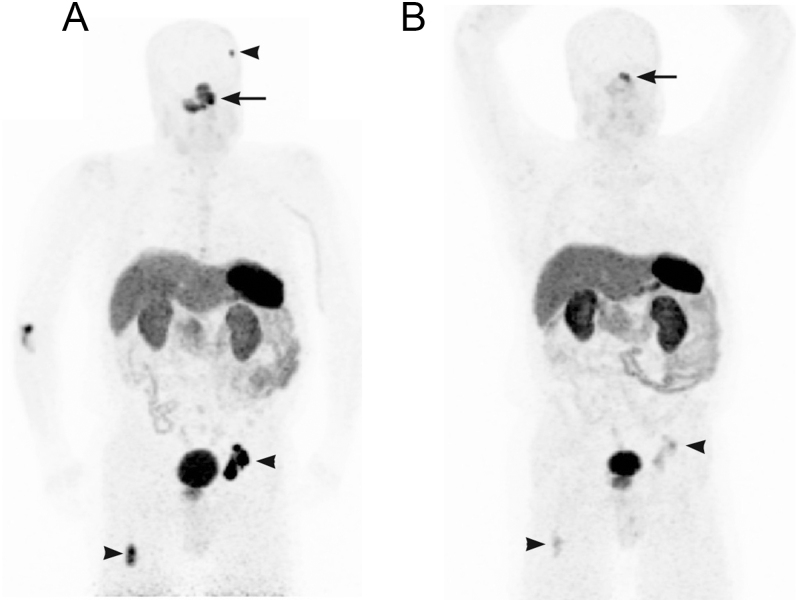
(A) Initial ^68^Ga-DOTATATE PET/CT obtained 7 days before initiation of PRRT demonstrating a somatostatin receptor-expressing infiltrative mass (arrow) in the central/left skull base, cavernous sinus, and parasellar region (SUVmax 18.0), with sclerotic metastases (arrowheads) in the left parietal bone (SUVmax 11.5), right proximal femur (SUVmax 18.4), and left acetabulum (SUVmax 33.3). (B) Following four cycles of ^177^Lu DOTATATE PRRT, repeat ^68^Ga-DOTATATE PET/CT 2 years following completion of PRRT demonstrated a partial metabolic response, with the primary (arrow) and osseous metastatic sites (arrowheads) demonstrating durable expression based on SUV measurements of target sites – left primary central/left skull base mass (SUVmax 12.5), left parietal bone (SUVmax 2.0), right proximal femur (SUVmax 5.6), and left acetabulum (SUVmax 4.9). Abbreviations: CT (computed tomography); Ga (gallium); Lu (lutetium); PET (positron emission tomography); PRRT (peptide receptor radionuclide therapy); SUV (standardized uptake value).

Following PRRT, the patient continued on maintenance monthly SSAs and zoledronic acid. Only after therapy with ^177^Lu DOTATATE was there a significant, sustained, progressive decrease in ACTH. While levels remain elevated, they are currently between 400 and 500 pg/mL (88–110 pmol/L) compared to 9,000–11,000 pg/mL (1,980–2,420 pmol/L) on SSA monotherapy and >40,000 pg/mL (>8,800 pmol/L) untreated. At the time of publication, the patient is 32 months post PRRT. MR brain demonstrates stable disease (−25.6% change from baseline) of the primary lesion per RECIST 1.1 size criteria, and ^68^Ga DOTATATE PET revealed a partial metabolic response with reduced tumor somatostatin expression (Fig. 3).

## Discussion

We present a case of sustained biochemical and structural improvement in a patient with metastatic PitNET following PRRT. Initially diagnosed with a presumably benign ACTH-secreting pituitary adenoma, he underwent more than a decade of multiple transsphenoidal resections, bilateral adrenalectomy, gamma knife irradiation, SSAs, and steroidogenesis inhibitors following recurrent and progressive tumor growth. Despite the above, he remained intermittently cushingoid with significant elevations in ACTH and was ultimately found to have osseous metastatic deposits. Following progression on traditional cytotoxic chemotherapy, he initiated ^177^Lu DOTATATE and has had tumoral shrinkage and biochemical improvement.

While the sequence from adenoma to metastatic disease is poorly understood, 60% occur in men, the ACTH-secreting subtype is overrepresented, and the clinical course is highly variable, with an average of 5.5 years from apparently benign to aggressive course (Burman *et al.* 2023). Metastases are typically found in the central nervous system and distant organs, including the liver and skeleton. Standard therapeutic options have varying results, and progression is common, leading to a search for alternative therapies.

In 2017, the phase 3 Neuroendocrine Tumors Therapy (NETTER-1) trial evaluating the efficacy and safety of ^177^Lu DOTATATE compared to high-dose octreotide in patients with advanced, progressive midgut NET demonstrated markedly longer progression-free survival (PFS) in the PRRT arm (Strosberg *et al.* 2017). The Food and Drug Administration (FDA) quickly approved PRRT for gastroenteropancreatic (GEP) NETs, and it has since been studied in NETs of multiple origins, albeit as an off-label therapy, which has limited eligibility to patients enrolled in experimental trials, particularly outside of the United States (US) (Muthukrishnan *et al.* 2021, Urso *et al.* 2023). Within the US, treatment may require a medical necessity letter and a peer-to-peer review with insurance carriers before initiation.

The first targeted use of radiolabeled SSAs in a primary pituitary pathology was in 2012. A 58-year-old female with an aggressive, giant prolactinoma complicated by severe neurological symptoms refractory to dopamine agonists and SSAs underwent four cycles of PRRT with ^111^In-DTPA-octreotide and had remarkable tumor shrinkage (Baldari *et al.* 2012). As of 2024, 20 cases of pituitary masses treated with PRRT were reported (Burman *et al.* 2023, Gandhi *et al.* 2024); however, just five of these cases involved metastatic PitNETs (Kovacs *et al.* 2013, Maclean *et al.* 2014, Bengtsson *et al.* 2015, Novruzov *et al.* 2015, Lin *et al.* 2021). A literature review between 2024 and 2025 demonstrated no additional cases. With the addition of our current case, the total number of reported patients with metastatic PitNETs who underwent PRRT is six (Table 1). Of note, an additional case report focused on immunotherapy in metastatic PitNET briefly notes (in a table) that a female patient underwent one cycle of PRRT as an eighth-line therapy, but as additional information was lacking, the patient has not been included (Majd *et al.* 2020).

**Table 1:** Literature review of PRRT in metastatic PitNET.

	Age*	Sex	Histopathology	Previous therapies	Bilateral adrenalectomy	Location metastases	PRRT (agent, cycles)	Response^†^	Side effects
Kovacs *et al.* (2013)	29	F	ACTH-secreting, Ki-67 10–25%	Surgery, RT	Yes	Hepatic, parotid	^90^Y DOTATOC, 2	Unassessed, patient deceased	None reported
Maclean *et al.* (2014)	85	M	NF, Ki-67 3%	Surgery, RT	No	Intraspinal, osseous	^177^Lu DOTATATE, 4	Tumor stability, isolated CR	Thrombocytopenia^‡^
Novruzov *et al.* (2015)	71	M	GnRH-secreting, Ki-67 ‘low’	Surgery, RT	No	Intraspinal, osseous	^177^Lu DOTATATE, 3	Tumor stability	None reported
Bengtsson *et al.* (2015)	46^§^	M	GH-secreting, Ki-67 60%	Surgery, RT, chemotherapy (TMZ), SSA	No	Intraspinal, hepatic	^90^Y DOTATATE, NR	Unassessed, patient deceased	None reported
Lin *et al.* (2021)	54	F	ACTH-secreting, Ki-67 unavailable	Surgery, RT, chemotherapy (CAPTEM), ICI	Yes	Hepatic, osseous, ovarian	^177^Lu DOTATATE, 4	Tumor stability^║^	None reported
Present case report	55	M	ACTH-secreting, Ki-67 5–10%	Surgery, RT, SSA, SI, chemotherapy (CAPTEM)	Yes	Osseous	^177^Lu DOTATATE, 4	Tumor shrinkage; stability	None reported

* Age at which PRRT was initiated.

^†^ Response based on post-PRRT imaging. If the patient was unable to complete post-therapy (early cessation, and death) they are listed as unassessed.

^‡^ Brief, 1 week with self-recovery ×1 week.

^§^ Age at which PRRT was initiated is unavailable; initial diagnosis of pituitary lesion.

^║^ ICI following PRRT was accompanied by a partial response with tumor shrinkage, which the authors suggested was a synergism between PRRT and ICI.

*Abbreviations:* CAPTEM, capecitabine and temozolomide; CR, complete response; F, female; ICI, immune checkpoint inhibitor; Lu, Lutetium; M, male; PitNET, pituitary neuroendocrine tumor; PRRT, peptide receptor radionuclide therapy; RT, radiation therapy; SI, steroidogenesis inhibitor; SSA, somatostatin analog; TMZ, temozolomide; Y, Yttrium.

Within this population, there was a male predominance (4M, 2F) with a median age of 56.6 years, which mirrors previous epidemiologic evaluations of uncommon adult pituitary tumors (Castellanos *et al.* 2022). Almost all patients developed metastatic deposits more than a decade after initial diagnosis, and 50% (*n* = 3) were ACTH-secreting, which parallels the disproportionate number of ACTH-secreting tumors resulting in aggressive disease compared to growth-hormone-secreting (*n* = 2) and non-functional (*n* = 1) tumors (Burman *et al.* 2023). Every patient had multiple surgical procedures or rounds of radiotherapy, the maximum combined number of which was more than ten. All patients with ACTH-secreting tumors underwent bilateral adrenalectomy complicated by Nelson syndrome. Of the patients (*n* = 4) who completed a full course of PRRT with 3 to 4 cycles, all had confirmed tumor stability, with 75% (*n* = 3) having significantly decreased tumor volume or complete resolution (*n* = 1) of isolated metastatic deposits. The only reported adverse reaction directly associated with PRRT (*n* = 1) was thrombocytopenia during cycle 1, week 4, that resolved without intervention. Given the somatostatin receptor expression in the anterior pituitary, several studies have evaluated the potential for anterior hypopituitarism following PRRT; however, in this population, given recurrent transsphenoidal resections and radiotherapy, secondary hypopituitarism is common and likely already present before PRRT (Elston *et al.* 2021).

While temozolomide-based chemotherapies remain the mainstay of systemic treatment for patients with metastatic PitNET, limited information is available for subsequent therapies following tumor progression. The revised 2025 guidelines from the European Society of Endocrinology suggest a second course of temozolomide, surgery, or radiotherapy following tumor progression on temozolomide, with later consideration for immune checkpoint inhibitors, targeted therapies, or PRRT (Raverot *et al.* 2025). Similarly, individual authors have proposed an algorithm for patients with aggressive and metastatic PitNET, with targeted agents like bevacizumab or immune-checkpoint inhibitors (ICI) ahead of PRRT (Iglesias 2023). While the author notes the strategy is based only on retrospective studies and clinical responses of small case series, they do not offer an explanation as to the order of pursued alternative therapies.

Given that PRRT was overall well-tolerated, with only one patient experiencing limited, self-resolving thrombocytopenia, and 67% (*n* = 4) of patients had tumor shrinkage or stability, PRRT should be considered earlier in patients with metastatic PitNET. Further studies are needed to determine if earlier implementation results in decreased morbidity and mortality. Finally, the inclusion of patients with metastatic PitNET in clinical trials will further guide providers in determining the order of novel second-line agents, including non-endocrine targeted therapies, immunotherapy, and PRRT.

## Declaration of interest

There are no relevant conflicts of interest that could be perceived as prejudicing the impartiality of the research reported for all authors (KIW, ZL, EAH, KKW, FPW, and TE).

## Funding

Research reported in this publication was supported by the National Institute of Diabetes and Digestive and Kidney Diseases of the National Institutes of Health under Award Number T32DK007245 (KIW).

## Author contribution statement

All authors (KIW, ZL, EAH, KKW, FPW, and TE) made individual contributions to authorship and were involved in the clinical diagnosis and management of this patient. KIW was responsible for the initial manuscript draft. ZL and KKW performed dosimetry analysis. All authors reviewed and approved the final draft.

## Informed patient consent for publication

Written informed consent for publication of their clinical details and/or clinical images was obtained from the patient.
